# Competencies for the provision of comprehensive medication management services in an experiential learning project

**DOI:** 10.1371/journal.pone.0185415

**Published:** 2017-09-26

**Authors:** Simone de Araújo Medina Mendonça, Erika Lourenço de Freitas, Djenane Ramalho de Oliveira

**Affiliations:** 1 Departamento de Farmácia Social, Faculdade de Farmácia, Universidade Federal de Minas Gerais, Belo Horizonte, Brazil; 2 School of Pharmacy, Regis University, Denver, United States of America; Waseda University, JAPAN

## Abstract

**Objective:**

To understand students’ and tutors’ perceptions of the development of clinical competencies for the delivery of comprehensive medication management services in an experiential learning project linked to a Brazilian school of pharmacy.

**Methods:**

An autoethnographic qualitative study was carried out based on participant observation, focus groups and individual interviews with students and tutors involved in an experiential learning project.

**Results:**

The study revealed the development of competencies related to the philosophy of practice, the pharmacotherapy workup of drug therapy and interprofessional relationships.

**Conclusions:**

The experiential learning project contributed to the professional development of pharmacy students in pharmaceutical care practice, pointing to its potential benefits for incorporation into professional pharmacy curricula.

## Introduction

Prompted by an emergent social need–drug-related morbidity and mortality [1,2]–the pharmacy profession is undergoing reform. To this end, pharmacists have been called to become competent in the provision of medication therapy management services, or more recently called comprehensive medication management (CMM) services [3–8]. These clinical services are the operationalization of the main principles of pharmaceutical care practice, which has been accepted as a new mission and model for professional pharmacy practice [4,9,10]. For pharmacists, this implies building therapeutic ties and developing a patient-centered practice [11]. This also means taking responsibility for the drug therapy-related needs of individuals and applying the pharmacotherapy workup throughout the whole care process [12]. This new role requires changes in the education of these professionals.

In countries like the United States of America–in which clinical pharmacy began its development in the first half of the twentieth century–, pharmacy education focuses on developing students’ clinical competencies and comprehensive pharmacotherapeutic knowledge [13–15]. However, as indicated by several authors [4, 9, 10], there are further competencies related to the philosophical and methodological principles of pharmaceutical care that also need to be incorporated into the pharmacy curriculum.

Ramalho de Oliveira [16] carried out an ethnographic study at the University of Minnesota (UofM), where pharmaceutical care was conceived, and within the health system where it was originally implemented. The study revealed shortcomings of the pharmacy curriculum in preparing professionals who felt encouraged and motivated to make this practice a reality. The author discussed the importance of students being exposed to the main components of the practice throughout the whole curriculum. Also, her study highlighted the importance of the culture of care in the curriculum, involving faculty members, students, and practitioners committed to the values and objectives defined by the philosophy of pharmaceutical care. After years of experience practicing pharmaceutical care, professionals from the same health system took part in an another educational study conducted by Losinski [17]. The results led to the definition of the curricular content and the core educational elements needed to prepare competent pharmaceutical care practitioners. Among the recommendations, the author emphasized the importance of experiential learning. Losinski noted that students should be engaged in practice from the beginning of the pharmacy program, since it is through practice that they develop an understanding of how to think, to be, and to act as a practitioner. Further exploring educational strategies to train professionals for pharmaceutical care, Freitas and Ramalho de Oliveira [18] conducted a study involving faculty members and students from that same university. The results found experiential learning to be a catalyst factor in developing critical thinking, a key element in training pharmaceutical care practitioners.

Consistent with this knowledge, the current accreditation standards and key elements for the professional program in pharmacy leading to the Doctor of Pharmacy degree in the USA [19] recommend integration of theory and practice, with placement of students in health services from the outset of the degree course. Thus, introductory and advanced pharmacy practice experience makes up around 30% of the curricula in schools of pharmacy in the USA. These guidelines have influenced scientific output in the field of pharmacy education, particularly in the USA, as outlined in the systematic literature review carried out by Mendonça et al [20]. According to this study, experiential learning is one of the strategies widely used to place students within health services [21–30]. Experiential learning theory (ELT), developed by the North-American researcher David Kolb [31], is one of the pedagogic approaches underpinning this educational strategy.

According to ELT, knowledge is constructed based on lived experiences. Kolb’s experiential learning cycle indicates that knowledge is derived from active experimentation of concepts. Hypotheses learned through concrete experiences are subsequently understood by reflection [31]. Therefore, educational programs seeking to promote integrative consciousness–the most complex level of development of human consciousness–should expose students to professional practice. Furthermore, students should be supported with instruments allowing reflection and promoting understanding of the elements experienced. Students should also be allowed to revisit practical situations and actively experiment with the newly developed concepts and hypotheses [31].

While countries such as the USA and Canada have a substantial experiential education component to their curricula, schools in most other countries do not have an extensive experiential component and are examining the utility and/or feasibility of adding such a component. In Brazil and other Latin American countries–where the clinical pharmacy movement is still incipient–there are other challenges. In Brazil, during the mid-twentieth century, the areas of clinical and toxicological analyses, bromatology, and the industrial production of medications all became part of pharmacy, with concomitant incorporation of knowledge and skills associated with these areas into the Bachelor of Pharmacy (BPharm) curriculum [32]. This situation calls for an even greater effort to transition from the traditional curriculum to the preparation of pharmaceutical care practitioners. Besides the need to incorporate the philosophy and patient care process proposed by pharmaceutical care practice, the BPharm curriculum in Brazil traditionally lags behind in the clinical and pharmacotherapeutic knowledge areas [32–35].

Pharmacists, faculty, and students involved with clinical practice and pharmacy education in Brazil have engaged in a movement for major changes in the professional curriculum [36,37]. Innovative educational approaches have been increasingly adopted in pharmacy professional programs [38]. There is also an increase in research in education for pharmaceutical care [16,32,39–41]. In light of the progress made in the field by other countries, it would be wise to leverage the knowledge gained, adapting and testing it for use in the Brazilian educational system.

In this context, the authors of the present study have identified a gap in the knowledge on experiential learning for training pharmaceutical care practitioners in the Brazilian BPharm curriculum. Consequently, the authors have developed an educational project in which the principles of experiential learning were applied in a project run in partnership with a public community clinic in Belo Horizonte, Brazil [42]. The objective of this study was to determine the contributions of this educational experience in the development of clinical competencies for the delivery of CMM services, based on the principles of pharmaceutical care practice, based on the perceptions of students and tutors involved.

## Methods

An autoethnographic qualitative study was carried out [43–45]. In order to ensure scientific rigor, the quality criteria of autoethnographic studies proposed by Le Roux [46] as well as the COREQ checklist [47] for qualitative studies in general were followed.

Data collection and interpretation were performed by one of the authors (SAMM)–who also served as the tutor for the students involved in the experiential project. The other authors (DRO and ELF) oversaw the data collection and interpretation of results. Autoethnography was elected as the best methodological option because one of the authors was immersed in the culture under investigation [44,45], actively interacting with the other study participants throughout the data collection and analysis process. According to Chang [44], autoethnography is a research methodology that combines elements of ethnography and narrative research. Under this method, the researcher scrutinizes autobiographic data in a critical, analytical and interpretive manner to identify cultural aspects of the experience under investigation.

### Curricular framework

The experiential learning project was run as part of a BPharm program in the state of Minas Gerais, Brazil. This academic program has a traditional curricular structure, spanning 10 semesters for the day course and 13 semesters for the evening course, both conferring the same degree. Students first undertake the basic cycle (theoretical-practical classes related to the natural sciences) and subsequently the professional cycle (theoretical-practical classes in pharmaceutical sciences). On the day course, students can elect an area in which to specialize including industry, bromatology, clinical and toxicological analyses or hospital pharmacy and health services. Students on the evening course cannot opt to specialize, following a pre-established curriculum including classes in all these different areas to qualify for their pharmacy degree. There are some classes in which students are exposed to different pharmacy settings, consisting predominantly of observational activities. At the end of the course, students embark on a compulsory practice internship within one drugstore plus a further placement in their area of specialization (day) or elected area (evening), such as clinical and toxicological analyses or the industrial production of medications. These practical placements are under the tutorship of the pharmacist in charge at the pharmacy in question, without parallel didactic activities at the university.

On this pharmacy program, an elective course is offered to introduce students to the professional practice of pharmaceutical care. This course includes background reading, practical exercises, reflections and discussions, providing students with the knowledge on the main philosophical and methodological elements of the practice. During this semester long course, enrolled students apply the principles learned to the care of a patient within their family/social circle. This elective course has been taught for 13 years, offering 20–25 seats per semester. The goal of the experiential learning project was to promote further development of pharmaceutical care competencies of students who took this introductory course.

### Structure of the experiential learning project

The project was directed by a faculty member associated with the pharmacy program. Three pharmacy graduate students served as tutors to the pharmacy students, overseeing the students in all their clinical activities [42]. Under a partnership established between the School of Pharmacy and the municipal public health system, students and tutors provided Comprehensive Medication Management Services (CMMS) grounded in the theoretical framework of Pharmaceutical Care Practice [3,4] in a primary healthcare clinic.

The pharmacy tutor:student ratio was 1:4 and 1:3, creating a fixed tie between two tutors and two pairs of students. The third tutor provided support when patient consultations occurred concurrently, so that the students were always accompanied by a tutor. One of the tutors (SAMM) oversaw didactic and reflection activities concerning the practice held at the university. The pre-requisites for students to take part in the experiential learning project were to have completed the pharmaceutical care introductory course and the two curricular pharmacology courses. Selected students had concluded, on average, 70% of the pharmacy degree course.

For six months, strategic planning was performed for provision of the CMM service in the primary healthcare clinic. Parallel to the planning stage, students and tutors had weekly meetings (2hrs/week) to study primary healthcare in the Brazilian public healthcare system, where they would practice, as well as the clinical protocols for the most common diseases managed in this scenario. The clinical activities within the health service took place during the ensuing six months, consisting of 4hrs of individual consultations and 2hrs of clinical meetings per week. In addition, a total of 2 hours per week of didactic reflexive activities on the practice were held in parallel, at the university.

The students’ primary responsibilities toward patients were to participate in their process of care, which involved working up their patients, documenting the appointments in the health record and participating in interprofessional clinic meetings. Since students had cared for 1–2 patients from their close social context in the previous pharmaceutical care introductory course, this experiential learning project was the students’ first clinical experience in a healthcare service. Thus, all the activities with patients and other professionals were performed under direct supervision of one of the tutors.

### Data collection

The first author acted as tutor in the experiential learning project [42,44,45]. This investigator kept field journals containing descriptive and reflective notes on observed situations, conducted informal interviews with students and with other tutors, and led tutor planning meetings. In addition to the first author’s field journals, descriptive and reflective journals maintained by all students and tutors throughout the project were collected and included in the data analysis [44]. The field journals were written and/or recorded in audio throughout the entire education experience period (12 months, average 4hrs per week).

A focus group discussion with all students and tutors was held at the end of the education experience [44]. A guide used to direct questions to the focus group encouraged participants to share their perceptions about learning in the practice setting. The duration of focus group was 1h 14min and it was held at the university.

A semi-structured in-depth interview was conducted by the first author with each pharmacy student at the completion of the education experience [44]. Use of an interview guide encouraged students to contextualize their perceptions about learning in the practice setting as part of their pharmacy training. The average time of interviews was 56 min and they were held at the university.

All interviews were conducted at the university and were audio-recorded. Audio-recorded data were transcribed verbatim by a profession transcriber. The first author checked transcription accuracy by comparing written data to the original recording, making any necessary corrections.

### Data analysis and interpretation

Data analysis occurred iteratively, with creation of provisory codes using NVivo, version 11 [48] a qualitative research software product. After the initial analysis, the three authors collaborated in the process of reassessing the initial codes, connecting or eliminating some codes, and defining the final themes of the study.

In this stage, analytical and interpretative memos were written to help elucidate the thematic units and categories structuring the results. Given the autoethnographic nature of the study, reflective statements written by the first authors and academic tutors that illustrated the phenomenon under investigation were identified [44]. The restricted number of study subjects involved in the project meant that data collection continued until all possible data were collected, rather than until saturation was reached [44,46].

### Ethical aspects

The present study was approved by the ethic committee for human research of the Federal University of Minas Gerais (process CAAE 25780314.4.3001.5140). Study participants gave written consent after being informed of all details of the study.

## Results and discussion

### Grasping philosophy in practice

Performing initial patient assessments in this setting sharpened participants’ listening skills, given that the life and medical histories of patients were not known to them beforehand, unlike their first patients. This contact with “real” patients promoted a sense of professionalism [49].

“I learned how to act like a health professional with a patient.” [Interview with student]

Structured documentation of clinical encounters was a required element of this project, serving for both didactic and clinical purposes. The structured documentation was didactic because it helped students learn the core elements of each stage of the care process. It also served a clinical purpose, assuring that all information required for the pharmacotherapy workup was collected [4]. Students reported that dealing with a structured documentation process was challenging for the first few consultations, when they were also getting to know the patient and striving to build therapeutic ties. It was all new to them.

“I learned to listen. Because it’s hard filling out that documentation system while the patient is saying other things. So, you think: ‘Oh my God, what am I going to do?’. They are speaking, and I can´t exactly say: ‘Hold on a second so I can fill this out’. No! You need to be patient. I think that´s it. I learned to be patient.” [Interview with student]

Being aware that they are living through a learning process helps students conduct the initial assessment more calmly, without feeling pressured to obtain all the data required during the time allotted for the first appointment.

“Can´t complete the initial assessment in a single visit? Let´s do it in two sessions! This requires patience on our part, too. And you should not think that you’re a failure because of it, because it’s a situation that really needs two sessions.” [Interview with student]

Recognition and validation of this feeling by the tutors allowed students to conduct the initial assessment with the quality required, putting into practice core elements of the philosophy of pharmaceutical care, such as patient-centeredness [4,50]. This context is conducive to accessing the patient’s medication experience, as emphasized by Ramalho de Oliveira [51]. Being centered on professional technical knowledge and having a paternalistic attitude towards patients are characteristics upheld by the traditional healthcare education model [50]. The same is found with traditional pharmacy degrees, leading to what was called the “pharmacists’ natural attitude”–an stance centered on pharmacologic knowledge and geared towards monitoring the correct use of medicines [51]. During participant observation we noticed that students struggled to break with this natural attitude.

“This evening during the consultation, I felt the patient´s uneasiness. The student began asking questions as if they suspected the patient had not been using the medications as instructed. And the more the student asked, the less the patient answered. It was obvious that the therapeutic relationship was not been fostered. I recognized that this is an important point for our reflections at the university.” [Academic tutor´s field journal]

Based on this observation, discussions about these experiences were included in the didactic reflexive meetings at the university. The team acknowledged the struggle and attempted to make the attitude towards patients more coherent with the practice philosophy.

The primary care setting allowed students to experience home visits, which helped strengthen the patient-centered practice grounded in the knowledge of the person as a whole, including their social and family background [50].

“Until that point I had not made visits. I had only conducted consultations at the clinic. I think it is really good, because you can better understand the person as a whole. Patients come to the health clinic all groomed and polished. When you visit their homes, they are leading a very different life. This is important for our work. (…) It helps us understand issues that may influence our assessment.” [Interview with student]

Even when home visits did not happen, students were stimulated to learn about patients’ family and social background. The discussions of clinical cases at the university were conducted from a biopsychosocial perspective [52]. This approach promoted a deeper understanding of the social and psychological factors influencing the process of care.

“Our discussions provided me with a clearer understanding of social and psychological factors. Before that I would only see the disease, the medications. And I was often unable to see beyond that.” [Student at focus group]

“I believe that by having discussions from this perspective [biopsychosocial], the student who was caring for the patient began to recognize other things and acted more effectively with both the patient and the health team.” [Tutor at focus group]

Notably, although the clinical discussions covered these aspects, students displayed a lack of basic knowledge of human and social sciences applied to health. This is an important gap in the curriculum revealed by this study.

### Developing skills to make decisions in clinical practice

The thought process needed to conduct the initial patient assessment had not been completely learned during classes taken at school, as identified in an interview with a student:

“I noted that I was lacking clinical experience and I needed more than one session to complete the initial assessment with my patients. Clearly, if you have more experience than a novice, you can think of more information to collect at the first appointment and you are able to identify some DTPs (drug therapy problems) right away. But those lacking experience, like me, need to consolidate this through more encounters with the patient.” [Interview with student]

Despite having studied contents such as biochemistry, anatomy, physiology, pathology, pharmacology, and pharmaceutical chemistry, among others, students felt unable to retrieve this knowledge to determine what needed to be further investigated with the patient. This gap is likely due to the fact that theory and practice are somehow disconnected in the curriculum. Students learned the theory, but few have had the opportunity to put it into practice. This disconnection can certainly hinder their learning process [31].

What pertinent information should be collected at the initial assessment? Students knew that they needed to ensure that each medication used by the patient was the most indicated, effective, safe and convenient for them. The pharmacotherapy workup had been studied and applied in the previous introductory pharmaceutical care course. Students could readily talk about this. However, a gap existed between knowing and doing:

“When I discussed the case with my tutor, it really opened up my mind. I realized that there were some basic things I was not addressing with the patient. I thought: ‘I understand the philosophy and what should be done. But the reasoning process isn’t working. It has gaps.’ Sometimes we have problems finding the answers because we don´t know how to reason properly. I think it was my case. And it is still true in my case, I think, because I´m still learning.” [Student at focus group]

The development of clinical skills to create hypotheses, resolve problems, and take rational decisions about pharmacotherapy was perceived differently by students. They knew the theory and had previous practical experience, yet further experience was needed for them to progress. As postulated by Kolb [31], the initial level of development of learners is characterized by a performative behavior and an identifying consciousness (acquisitive level). According to experiential theory, knowledge is produced by learning the subject through concrete experience and understanding it through critical analysis (reflective observation and abstract conceptualization) and active experimentation. In the integrative level of development, the consciousness is structurally integrated, bringing together actions, operations and meanings in a holistic manner. The individual is able to identify their own competencies and educational needs [31]. Several cycles of learning and comprehension are needed to take the learner from the acquisitive level to the integrative level of development and this was becoming clear to the students.

“Clinical decision is still very hard for me. I think this is developed over a long period, isn’t it? And we have only a few months of clinical experience. It seems a lot compared to the other students who didn´t take part in this project. But, on the other hand, it´s still nothing.” [Interview with student]

The participant students are aware they are undergoing a process, acquiring initial clinical experiences, building the foundation of their clinical knowledge [4]. The tutor reflects on this point in the field journal:

“I feel like a professional undergoing constant training, since I never had an ‘official’ educational process to provide pharmaceutical care. As someone who has been in the same position as these students are now, seeking extracurricular opportunities to develop clinical competencies, I understand their anguish, particularly given the absence of opportunities to practice safely while furthering their learning. I feel they are anxious to know everything right now. And they are only in the field for a few months. Actually, much longer is needed. This process should take place in a spiral mode throughout the pharmacy program. (…) It’s good to know they have begun their training, they are perceiving their educational needs, and can go forward building their career paths. They are going to be much better than I have managed to be up until now. This motivates me to create further opportunities for more students.” [Academic tutor field journal]

This highlights the importance of creating more opportunities for students to gain clinical experiences during their pharmacy degree via the inclusion of multiple clinical experiences throughout the curriculum. In addition, having a safe space for reflection and discussion of the learned knowledge–whether through formal courses or study groups–seems to be a key element of an effective learning process.

#### Retrieving information to identify and resolve drug-therapy problems

The application of the pharmacotherapy workup helped students to organize the knowledge acquired prior to the project and to seek and apply new knowledge during the initial assessment of patients.

Students reported major difficulties retrieving technical-scientific information to address clinical cases at the beginning of the experiential project. They had little familiarity with drug information and pharmacotherapy handbooks, clinical protocols and therapeutic guidelines.

“I already knew I´d have to look for protocols… My problem was how exactly to find these protocols.” [Interview with student]

This shows the weaknesses of an educational model that focuses on the transmission and retention of knowledge while overlooking the fact that learning does not occur through cognitive processes alone. As held by Kolb [31], learning involves theoretical and experiential knowledge. It should involve not only cognition, but also affection, perception and action.

“I read the whole therapeutic guideline on diabetes to take care of that patient living with diabetes. I knew he was awaiting to hear my position on his treatment and this forced me to study. Today I feel I have more knowledge than I had before.” [Interview with student]

As they progress practicing the pharmacotherapy workup and become more experienced in handling the literature, doubts arise over which choices to make in different scenarios. It is the uncertainty of clinical practice forcing them to grow and deal with reality, with its risks and the inherent responsibility.

“Sometimes I get hung up in a detail. As a result I may be unable to implement the intervention I was planning on. So, finding the literature is one thing, applying this scientific information in the patient´s case is another, which in turn hinges on clinical experience.” [Student at focus group]

At this point, the didactic reflective meetings with tutors proved pivotal in assuring quality care delivered to patients and student learning. Moreover, students emphasized they learned from observing how experienced pharmacists based their decisions on the best available evidence, yet also dealt with uncertainties.

#### Finding gaps in knowledge to care for patients

Besides the cognitive structure and skills to manage quick reference information sources, there seems to be some basic knowledge that needs to be incorporated into the training of pharmacists for clinical practice. Participant observation with students during the periods of initial assessment and in clinical case discussions revealed a lack of knowledge on physiopathology, semiology and pharmacotherapy. The students expressed this perception during individual interviews and focus group:

“I´ve been familiar with the databases for some time now. And I´ve worked with researchers. But even still, I have problems when it comes to studying clinical cases and resolving things. It´s not something that comes easily to me. I read the protocol and I think: ‘OK. And now what?’. It´s like there´s something missing. ‘This isn´t what I´m looking for at all. I need more’. And I just don´t know what that more is. It´s really agonizing…” [Student at focus group]

“I got frustrated and thought: ‘This isn´t right… I love pharmacology. I have to find out’. But I checked out the book and I didn´t find the answer. It wasn´t for lack of trying. When I thought of checking another information source on drugs I thought: ‘No, the problem is that I don´t know this disease.’ So I checked out the physiopathology book and tried to understand the disease.” [Student at focus group]

Thus, before moving on to quick reference sources on pharmacotherapy and drugs–resources extensively used in clinical practice by more experienced pharmacists–the students needed to make use of textbooks. This aspect had an impact on the pace of the experiential learning project, meaning that more time was required for students to study and progress safely in the care process. The case load had to be reduced and more individual meetings with the academic tutor had to be arranged to discuss the clinical cases encountered in practice.

At the end of the semester, the students noted that concrete experiences promoted a more effective learning process.

“Nowadays, if I have to see a patient with diabetes, I´d know that I´d have to check the vitamin B12 levels on patients using metformin. I´d know the importance of investigating how the patient is administering insulin. These seem silly things, but when you´ve never seen anyone doing that, it´s hard. You have to do it for the first time. I think this is important for me. I have a little more knowledge than I had before and this type of knowledge can only be gained through clinical practice… There´s no way around it. It’s little help the professor up there explaining how to use the inhaler device. It doesn´t stick… When you see the patient, you’ll say: ‘What did the professor say about it?’. It´ll raise flags, and… I’ll have to study again. It´s through practice that we learn.” [Interview with student]

### Establishing interprofessional relationships

Implementing interventions planned based on case studies improved students’ ability to negotiate with patients and improved their skills to collaborate with other healthcare professionals. In the process of establishing interprofessional relationships, students experienced initial feelings of fear and insecurity that can be attributed to the fact that this was their first contact as professionals with other members of the health team.

“Having to take part in the team meeting and discuss the patient´s case helped me mature in terms of having the courage to speak my mind. I studied, I know it, I can speak my mind. This changed my way of thinking and working.” [Interview with student]

The experiential learning project exposed students’ hesitance to working as part of a team, as raised by some students in the focus group:

Student A: “I feel a lack of confidence in myself. I feel that I´m unable to change something that a physician did [emphasis on the word physician], to make a suggestion, particularly because I am a student. I am reluctant to get into a discussion with the physician. Am I knowledgeable enough to discuss with her? She´s got years of clinical experience in the health system. How am I going to discuss with her?”

Student B: “And what if you think you will learn with them?”

Student A: “Right, but then the person has to be open to that. And she is a bit harsh. She listens to you. But she responds like this, raising an eyebrow, in a superior tone: ‘I understand what you are doing, but I understand what I´m doing really well. You´re not going to change what I do or change my opinion’. So it´s hard.”

Student C: “But it´s really positive that we go through this before graduation because real life is like this.”

Student A: “Definitely! I don´t consider this a bad experience. I see her [referring to the physician] as a professional who, despite being older and being longer in the service, is somewhat resistant. They have probably seen many professionals come and go… So, I see it like a person who is more resistant to team working.” [Students at focal group].

Several planned interventions that required the approval of other healthcare professionals were not implemented and they were a source of frustration for students. However, some students understood that those situations were part of building a shared responsibility amongst different healthcare professionals and they also acknowledged the constraints currently imposed by the health system.

“I took some time on a case of one of the patients, trying to get her to do a glycemic map to determine whether she needed regular insulin. When I got it, I took it along to the team meeting. I said she needed regular insulin. The physician just increased her NPH insulin dose… [pause]. But then, we see that it´s just the way it is. It´s a team effort. He [the physician] listened to me, recognized that the patient´s health problem was not well-controlled, and made another decision. And I followed up by monitoring the patient´s outcome.” [Interview with student]

Tutor support was highlighted as fundamental in tackling difficult situations during the activities at the clinic. In addition, the meetings at the university, in which students shared similar experiences and also successful experiences in collaboration with the health team, encouraged them to persist in their efforts to help patients.

“Many complained, but the physician in my team was excellent. She was really important in our journey. I showed up at many scheduled healthcare team meetings that never took place. One day she said to me: ‘I can´t believe you came again… Today I´ll sit by your side. I´m available to listen to you.’ I earned her trust!” [Interview with student]

During this experiential learning project, pharmacy students established interprofessional relationships and became members of the healthcare team. The students strongly emphasized the need to collaborate with physicians so that they could influence the patients’ pharmacotherapy and implement their care plans. Also, positive aspects were highlighted regarding the relationship with the nursing team and community health workers that helped particularly with the referral of patients to the CMM service. The incorporation of interprofessional educational strategies into professional curricula has been recommended as a strategy to consolidate interprofessional collaborative practices in health systems [53,54]. Experiential learning programs involving students, faculty members and practitioners from different disciplines can help train professionals that are better prepared for collaborative practice after graduating, consolidating this practice within health systems [55,56].

## Conclusions

This experiential learning project allowed students to achieve good levels of competency in applying the philosophical principles of pharmaceutical care by managing real patients within a primary care setting. This experience furthered students’ understanding and their ability to apply the pharmacotherapy workup in caring for real patients. In addition, the participating students refined their skills to retrieve technical-scientific information about medications. The participants developed a critical notion of the gaps in their knowledge on the clinical foundations of pharmaceutical care in their education to date, allowing them to seek strategies to bridge this educational gap.

Another evident outcome was the development of competencies for interprofessional relationships. The students had to deal with their emotions, such as fear and insecurity with regards to interacting with other health professionals. They were exposed to both successful relationships as well as difficulties establishing collaborative practice.

The students achieved good levels of development in each of the competencies outlined above, as predicted under the experiential learning theory proposed by Kolb.

Limitations: This study was limited to one of 430 BPharm programs currently available in Brazil. Thus, further educational studies are warranted exploring experiential learning projects in other pharmacy schools. Moreover, it should be considered that only a small portion of the total number of pharmacy students was exposed to the educational experience under investigation in this study. We believe that all undergraduate students from all pharmacy schools must have an experiential learning component in their curriculum. The benefits of such innovation, as has been pointed out in the literature and evidenced in this study, may outweigh the costs involved.

Implications for education: the results of this study point to the advantages of implementing experiential learning projects in pharmaceutical care during the course of the BPharm degree, promoting continuous and sustained professional development. Although a sufficient number of qualified faculty members are needed to carry out the tutorship role in these programs, this study reveals a possible transition model in which students with advanced clinical experience (graduate students, extending to residents and last year pharmacy students) can act as tutors of novice students. The need to incorporate basic clinical knowledge (physiopathology, semiology and pharmacotherapy), as well as human and social sciences applied to health, into the BPharm curriculum was also evident.

Implications for research: this study showed the potential of qualitative studies conducted by faculty members to study the educational culture of which they are part, promoting permanent education of professors through reflective practice.
